# Association between immune checkpoint inhibitor medication and uveitis: a population-based cohort study utilizing TriNetX database

**DOI:** 10.3389/fimmu.2023.1302293

**Published:** 2024-01-09

**Authors:** Hou-Ting Kuo, Chia-Yun Chen, Alan Y. Hsu, Yu-Hsun Wang, Chun-Ju Lin, Ning-Yi Hsia, Yi-Yu Tsai, James Cheng-Chung Wei

**Affiliations:** ^1^ Department of Ophthalmology, China Medical University Hospital, China Medical University, Taichung, Taiwan; ^2^ Department of General Medicine, China Medical University Hospital, Taichung, Taiwan; ^3^ Department of Medical Research, Chung Shan Medical University Hospital, Taichung, Taiwan; ^4^ School of Medicine, College of Medicine, China Medical University, Taichung, Taiwan; ^5^ Department of Optometry, Asia University, Taichung, Taiwan; ^6^ Institute of Medicine, Chung Shan Medical University, Taichung, Taiwan; ^7^ Department of Allergy, Immunology and Rheumatology, Chung Shan Medical University Hospital, Taichung, Taiwan; ^8^ Institute of Integrated Medicine, China Medical University, Taichung, Taiwan

**Keywords:** uveitis, ICI medication, immune checkpoint inhibitors, ocular adverse events, risk, TriNetX, United States

## Abstract

**Objective:**

To explore the associations between the use of immune checkpoint inhibitors (ICIs) and the risk of developing uveitis among cancer patients.

**Methods:**

Cancer patients who received ICI therapy and a comparison group of cancer patients who did not receive ICI therapy were retrospectively recruited from the TriNetX electronic heath-record registry. The outcome of interest was the development of new-onset uveitis. Propensity score matching based on a 1:1 ratio was conducted in order to reduce bias. Multi-variate cox proportional hazard models and Kaplan Meier method were also utilized to assess for the risk of uveitis among cancer patients who received ICI therapy.

**Results:**

71931 cancer patients (54.7% male; 76.5% white; mean age at index 63.6 ± 12.2 years) who received ICI treatment (ICI group) and 71931 cancer patients (54.7% male; 77% white; mean age at index 63.5 ± 12.4 years) who never received ICI (comparison group) were recruited. Associated Kaplan-Meier curves showed significantly increased uveitis risk among the ICI group for all follow-up years (p<0.001). The risk of uveitis was also higher among the ICI group during the 144-month follow-up period with a hazard ratio (HR) of 2.39 (95% CI: 2.07-2.75). Increased risk for specific uveitis diseases, such as iridocyclitis, chorioretinal inflammation, retinal vasculitis, unspecified purulent endophthalmitis, pan-uveitis and sympathetic uveitis were found. Subgroup analysis demonstrated an elevated hazard ratio for the development of uveitis among ICI recipients, spanning individuals below the age of 65 as well as those aged 65 and older. The elevated hazard ratio for uveitis development among ICI recipients was also observed across all genders, among those of white and Asian ethnicities, those with smoking history, and those with comorbid conditions such as hypertension and dyslipidemia, in comparison to their non-ICI counterparts. An additional subgroup analysis on monotherapy versus combinatory ICI regimens was also conducted. Individuals who received monotherapy from the class of anti-PD-1 (HR:1.98 [CI: 1.65-2.37]) and anti-CTLA-4 (HR:5.86 [CI:1.99-17.24]) exhibited elevated hazard ratios for uveitis development compared to their non-ICI comparators. Those exposed to combinatory ICI regimens, specifically a combination of anti-PD-1 and anti-CTLA4 (HR: 5.04 [CI:3.55-7.16]), showed increased hazard ratios for uveitis development compared to their non-ICI comparators. In contrast, individuals exposed to a combination of anti-PD-1 and anti-PD-L1 (HR: 2.47 [CI:0.81-7.50]) did not demonstrate an increased risk for uveitis compared to their non-ICI comparators.

**Conclusion:**

A significantly increased risk for uveitis diseases was found among the ICI group from the first year of follow-up. Increased awareness should be promoted on the occurrence of uveitis among cancer patients receiving ICI therapy.

## 1 Introduction

Uveitis is a leading cause of visual impairment worldwide (1) and causes for it can be broadly subdivided into infectious and noninfectious etiologies (2). One such noninfectious etiology is drug-induced uveitis. Drug-induced uveitis is considered to be a rare cause of uveitis, with previous estimates ranging around 0.5% of global uveitis cases (3). Recently, increased attention has been placed on drug-induced uveitis associated with immune checkpoint inhibitors (ICI). Immune checkpoint inhibitors (ICI) are novel cancer therapies that work by utilizing the immune system in order to target tumor cells. Its therapeutic action –based on influencing the immune system, has also been reported to cause unintended inflammatory-related side effects. Such side effects are termed immune-related adverse events (IRAEs). Ophthalmologic IRAEs, including uveitis are theoretically rare as the eye is considered to be an immune-privileged site (4). Given such rarity, a large population-based study would be required to quantify the actual uveitis risks among ICI users properly.

To address the knowledge deficits, population-level data was obtained from the TriNetX database. This multi-institutional platform houses de-identified health insurance claims and electronic health records of over a hundred million patients across various countries and institutions. These institutions encompass hospitals as well as primary care units. The dataset also includes information from insured and non-insured patients. The extensive nature of the TriNetX dataset presents a valuable opportunity to address existing knowledge gaps, particularly in the understanding of drug-induced uveitis secondary to ICI. Previous studies have successfully employed the TriNetX to investigate uveitis risks associated with different conditions (5). Our study aimed to explore the risk of uveitis development among cancer patients receiving ICI therapy using the TriNetX database.

## 2 Methods

### 2.1 Study design and data collection

This population-based, retrospective-matched cohort study utilized the TriNetX analytics platform. Data from the TriNetX includes basic demographics (such as age and gender), diagnoses (coded using the International Classification of Diseases, Tenth Revision, Clinical Modification, ICD-10-CM codes), procedures (coded using Healthcare Common Procedure Coding System (HCPCS)) and medications (coded using RXNORM). The TriNetX database has been deemed compliant with the Health Insurance Portability and Accountability Act (HIPPA) as well as certified to the ISO 27001:2013 standard. In terms of compliance with HIPPA, all data from the TriNetX has been formally attested by an accredited auditor as defined under Section §164,514(b)(1) of HIPPA. Additionally, TriNetX maintains its own Information Security Management System (ISMS), which further ensures the integrity of its privacy protection. This study was approved by Chung Shan Medical University Hospital Institutional Review Board (IRB NO: CS2-21176).

### 2.2 Study participants and eligibility criteria

A flow chart that outlines our cohort construction was depicted in **Supplementary Figure 1**. Study participants who were aged 20 years or older, diagnosed with cancer of all causes and received immune checkpoint inhibitors (ICI) from January 1^st^, 2011 to December 31^st^, 2022 were recruited. Those with cancer of all causes were identified from the database with the relevant ICD 10 codes (ICD10: C00-C96). Those who received ICI were also recruited based on the associated RxNORM and HCPCS codes. The immune checkpoint inhibitors of interest included: Nivolumab (RXNORM:1597876, HCPCS:J9299); Ipilimumab (RXNORM:1094833, HCPCS:J9228); Avelumab (RXNORM:1875534, HCPCS:J9023); Pembrolizumab (RXNORM:1547545, HCPCS:J9271); Atezolizumab (RXNORM:1792776, HCPCS:J9022); Cemiplimab (RXNORM:2058826, HCPCS:J9119); Dostarlimab (RXNORM:2539967, HCPCS:J9272); and Durvalumab (RXNORM:1919503, HCPCS:J9173). Four of the ICI recruited (i.e., Nivolumab, Cemiplimab, Dostarlimab and Pembrolizumab) belong to the class of programmed death-1 (PD-1) inhibitors, while the other three ICI (i.e., Atezolizumab, Avelumab, and Durvalumab) belong to the class of programmed death ligand-1 (PD-L1) inhibitors. Only one of the ICI medications (Ipilimumab) investigated belonged to the cytotoxic T-lymphocyte antigen 4 (CTLA-4).

Cancer patients (based on ICD-10: C00-C96) who never received immune checkpoint inhibitors (based on RxNORM codes) were defined as the comparison cohort (see **Supplementary Table 1**). The index date for the ICI group was defined as the date from which the first prescription for ICI was given. The index date for the control group was based on a randomized date from 2019 to 2022.

Previous studies have established links between uveitis and certain conditions (6–16). To reduce confounding, our study (see **Supplementary Table 2**) excluded those diagnosed with: Human immunodeficiency virus (ICD-10-CM = B20, R75, Z21, B97.35), syphilis (ICD-10-CM: A51.0, A52.75, A52.7, D89.89), Sarcoidosis (ICD-10-CM: D86), autoimmune hepatitis (ICD-10-CM: K75.4), parapsoriasis (ICD-10-CM: L41), psoriasis (ICD-10-CM: L40), systemic lupus erythematosus (ICD-10-CM: M32), rheumatoid arthritis (ICD-10-CM:M05, M06), juvenile arthritis (ICD-10-CM: M08), chronic kidney disease (ICD-10-CM: N18), and Behcet’s disease (ICD-10-CM: M35.2).

In terms of baseline characteristics and comorbidities, records were obtained from one year before the index date. Baseline demographics of interest included: age, gender, ethnicity and the setting of the health-care utilization. Baseline comorbidities of interest included: smoker status (ICD-10-CM: Z72.0), hypertension (ICD-10-CM: I10-I16), dyslipidemia (ICD-10-CM: E78), coronary artery disease (ICD-10-CM: I20-I25) and cerebrovascular disease (ICD-10-CM: I60-I69).

A 1:1 cohort was generated with patients from the ICI group and the non-ICI comparison group and matched by propensity score using the built-in function from the TriNetX platform. Variables considered by our propensity score-matching analysis included: sex, race, comorbidities and medical utilization.

### 2.3 Outcomes and variables

We sought to investigate incident uveitis following exposure to ICI among cancer patients. The primary endpoint was composed of uveitis conditions occurring up to the end of the study period (December 31^st^, 2022) after the index date. Consistent with methods validated by previous studies (5), we defined incidence uveitis cases based on the following criteria:

1. An ICD-10 diagnostic code relating to specific uveitis-related diseases that has been validated in previous studies (5, 17)
2. At least two healthcare visits separated by at least one week apart with an ICD-10 code indicating uveitis.

All patients were followed up from the index date until the earliest occurrence of the outcome of interest (e.g., uveitis) or drop-out from the study for any reason. Additionally, participants with uveitis diagnosis prior to the index date were excluded. The uveitis conditions of interest were defined by the ICD-10 codes extracted from the electronic records (refer to **Supplementary Table 3**) and included the following: Iridocyclitis [ICD-10=CM=H20], Chorioretinal inflammation [ICD-10-CM=H30]; Retinal vasculitis [H35.06]; Unspecified purulent endophthalmitis [H44.0]; Panuveitis [H44.11]; Sympathetic uveitis [H16.24]; Vogt-Koyanagi-Harada disease (VKH) [H20.823]; and Harada’s disease [H30.819]. These endpoints are selected based on their validation in prior studies (5, 7) and are broadly used among various academic tertiary centers in the United States (18). Additionally, the inclusion of VKH and Harada’s disease was motivated by their established association with ICI, making them particularly relevant to the focus of this study.

### 2.4 Statistical analysis

TriNetX platform was used to perform all of our statistical analysis. Standardized mean differences (SMD) was used to ascertain the distribution balance among our baseline variables. Variables with SMD values of less than 0.1 were considered well-matched. Cox proportional hazards regression analysis was also utilized to analyze the matched cohorts. Hazard ratios and 95% confidence intervals (95% CI) were reported in this analysis. Kaplan-Meier method and log-rank test were also utilized to calculate the incidence for uveitis. Statistical significance was defined as two-sided p-value <0.05.

## 3 Results

### 3.1 Baseline comorbidities of the study participants

After propensity score matching, the study recruited 71931 cancer patients (54.7% male; 76.5% white; mean age at index date 63.6 ± 12.2 years) who received ICI treatment and 71931 cancer patients (54.7% male; 77% white; mean age at index date 63.5 ± 12.4 years) who never received ICI (see **Table 1**). The baseline details of interest included demographics (e.g., age, gender, ethnicity), comorbidities and health-care utilization settings from both the ICI group and the non-ICI comparison group. Such details were obtained before and after propensity score matching (see **Table 1**). The two groups were found to be well matched in terms of distribution by baseline factors (SMD<0.1).

**Table 1:** Demographic characteristics of immune checkpoint inhibitors (ICI) and non-immune checkpoint inhibitors (ICI).

	Before PSM matching		p	SMD	After PSM matching		p	SMD
	ICI N = 71931	Non-ICI N = 4074752			ICI N = 71931	Non-ICI N = 71931		
Age, Mean ± SD	63.6 ± 12.2	60.9 ± 14.0	<0.001	0.204	63.6 ± 12.2	63.5 ± 12.4	0.471	0.004
Sex								
Female	30879 (42.9)	2104822 (51.7)	<0.001	0.175	30879 (42.9)	30989 (43.1)	0.558	0.003
Male	39326 (54.7)	1894281 (46.5)	<0.001	0.164	39326 (54.7)	39360 (54.7)	0.857	0.001
Race								
White	54992 (76.5)	2982403 (73.2)	<0.001	0.075	54992 (76.5)	55376 (77.0)	0.017	0.013
Black or African American	5932 (8.2)	344226 (8.4)	0.055	0.007	5932 (8.2)	5934 (8.2)	0.985	<0.001
Asian	2401 (3.3)	106162 (2.6)	<0.001	0.043	2401 (3.3)	2310 (3.2)	0.178	0.007
Comorbidities								
Tobacco use	7676 (10.7)	74184 (1.8)	<0.001	0.372	7676 (10.7)	7480 (10.4)	0.092	0.009
Hypertension	31628 (44.0)	620493 (15.2)	<0.001	0.663	31628 (44.0)	31926 (44.4)	0.114	0.008
Dyslipidemia	21850 (30.4)	507495 (12.5)	<0.001	0.448	21850 (30.4)	21916 (30.5)	0.705	0.002
Coronary artery disease	12329 (17.1)	171809 (4.2)	<0.001	0.428	12329 (17.1)	12325 (17.1)	0.978	<0.001
Cerebrovascular disease	6968 (9.7)	90974 (2.2)	<0.001	0.319	6968 (9.7)	6939 (9.6)	0.796	0.001
Medical utilization								
Ambulatory	66136 (91.9)	2217356 (54.4)	<0.001	0.935	66136 (91.9)	66160 (92.0)	0.816	0.001
Emergency	17369 (24.1)	283678 (7.0)	<0.001	0.488	17369 (24.1)	17460 (24.3)	0.575	0.003
Inpatient Encounter	29321 (40.8)	316320 (7.8)	<0.001	0.834	29321 (40.8)	29337 (40.8)	0.932	<0.001

SMD, standardized mean difference.

### 3.2 Risk of uveitis among the Immune complex inhibitor recipients and comparison group in terms of follow-up duration

The risk of developing uveitis diseases among the ICI group compared to the comparison group was the primary outcome, and this was assessed at specific follow-up intervals up to 1-year, 2-year, 5-years and 12-years following the index date (see **Table 2**). These specific follow-up intervals from baseline were evaluated as we wanted to assess both the short-term and long-term risk of uveitis. Similar studies in the past, with the risk of uveitis as the primary outcome, have also employed similar intervals and frequency of follow-ups (5). We also performed a risk-stratification analysis to investigate potential factors associated with uveitis development among recipients of immune checkpoint inhibitors and compared them to non-immune checkpoint inhibitors users at corresponding follow-up duration of interest.

**Table 2:** Risk of uveitis exposed to immune checkpoint inhibitors (ICI) compared with non-immune checkpoint inhibitors (ICI).

	No. of uveitis		HR (95% C.I.)
	ICI N = 71931	Non-ICI N = 71931	
All duration			
Uveitis (All)	486	373	2.39 (2.07–2.75)
Iridocyclitis	343	243	2.59 (2.18–3.09)
Chorioretinal inflammation	94	49	3.71 (2.57–5.37)
Retinal vasculitis	10	10	5.64 (1.54–20.62)
Unspecified purulent endophthalmitis	92	103	1.58 (1.18–2.12)
Panuveitis	34	10	8.06 (3.51–18.51)
Sympathetic uveitis	10	10	7.57 (1.55–36.92)
Vogt-Koyanagi syndrome, bilateral	10	0	NA
Harada’s disease, unspecified eye	0	0	NA
1 year			
Uveitis (All)	312	105	3.27 (2.62–4.08)
Iridocyclitis	228	62	4.03 (3.04–5.34)
Chorioretinal inflammation	55	18	3.36 (1.97–5.73)
Retinal vasculitis	10	10	5.74 (0.67–49.23)
Unspecified purulent endophthalmitis	53	33	1.77 (1.15–2.74)
Panuveitis	21	10	11.18 (2.62–47.71)
Sympathetic uveitis	10	10	5.46 (0.64–46.74)
Vogt-Koyanagi syndrome, bilateral	10	0	NA
Harada’s disease, unspecified eye	0	0	NA
2 years			
Uveitis (All)	402	174	2.87 (2.40–3.43)
Iridocyclitis	288	105	3.37 (2.70–4.22)
Chorioretinal inflammation	76	22	4.24 (2.63–6.83)
Retinal vasculitis	10	10	9.37 (1.14–76.75)
Unspecified purulent endophthalmitis	71	61	1.50 (1.07–2.12)
Panuveitis	25	10	5.87 (2.24–15.36)
Sympathetic uveitis	10	10	10.26 (1.28–82.50)
Vogt-Koyanagi syndrome, bilateral	10	0	NA
Harada’s disease, unspecified eye	0	0	NA
5 years			
Uveitis (All)	475	295	2.48 (2.14–2.88)
Iridocyclitis	336	191	2.70 (2.25–3.23)
Chorioretinal inflammation	92	34	4.19 (2.80–6.26)
Retinal vasculitis	10	10	13.78 (1.73–109.80)
Unspecified purulent endophthalmitis	90	88	1.62 (1.20–2.18)
Panuveitis	33	10	8.44 (3.49–20.39)
Sympathetic uveitis	10	10	12.35 (1.55–98.20)
Vogt-Koyanagi syndrome, bilateral	10	0	NA
Harada’s disease, unspecified eye	0	0	NA

If the patient’s count is 1–10, the results indicate a count of 10.

NA, Not applicable.

At the 1-year timepoint following the index date, an overall increased risk for uveitis was found (HR: 3.27 [CI: 2.62-4.08]). Furthermore, we also performed a risk-stratification analysis that focused on potential factors linked to uveitis development among ICI recipients at the one-year follow-up. We observed elevated hazard ratio (HR) values for the development of uveitis among ICI recipients who were aged less than (HR: 3.73 [CI: 2.73-5.10]) and greater (HR: 3.24 [CI: 2.33-4.52]) than 65 years of age; female (HR: 2.84 [CI: 2.08-3.86]); male (HR: 3.34 [CI: 2.45-4.56]); White ethnicity (HR: 3.79 [CI: 2.93-4.90]); smoker (HR: 2.01 [CI:1.02-3.98]); and possessing hypertension (HR: 1.94 [CI: 1.30-2.89]), dyslipidemia (HR: 2.26 [HR: 1.39-3.68]) and cerebrovascular comorbidities (HR: 3.94 [CI:1.30-11.99]) compared to their respective non-ICI comparators (see **Supplementary Table 4**).

The uveitis diseases that had an increased risk of association with ICI usage at the 1-year time point following the index date were iridocyclitis (HR: 4.03 [CI: 3.04-5.34]); chorioretinal inflammation (HR: 3.36 [CI: 1.97-5.73]); unspecified purulent endophthalmitis (HR: 1.77 [CI: 1.15-2.74]) and pan-uveitis (HR: 11.18 [CI: 2.62-47.71]) (see **Table 2**).

At the 2-year timepoint, an overall increased risk for uveitis was found (HR: 2.87 [CI: 2.40-3.43]). Furthermore, we also performed a risk-stratification analysis on potential factors associated with uveitis development among users of immune checkpoint inhibitors at the two-year follow-up time point. We observed elevated hazard ratio (HR) values for the development of uveitis among ICI users who were aged less than (HR: 3.50 [CI: 2.70-4.53]) and greater (HR: 2.77 [CI: 2.13-3.60]) than 65 years of age; female (HR: 2.89 [CI: 2.22-3.78]); male (HR: 3.22 [CI: 2.49-4.17]), White ethnicity (HR: 3.41 [CI: 2.76-4.29]); positive smoker status (HR: 1.87 [CI: 1.01-3.46]); and had comorbidities of hypertension (HR: 1.60 [1.01-3.46]), dyslipidemia (HR: 1.67 [CI: 1.15-2.24]), and cerebrovascular disease (HR: 2.66 [CI: 1.08-6.57]) (see **Supplementary Table 5**) compared to their respective non-ICI comparators. The involved uveitis diseases that had an increased risk of association with ICI usage at the 2-year time point were iridocyclitis (HR: 3.37 [CI: 2.70-4.22]); chorioretinal inflammation (HR: 4.24 [CI: 2.63-6.83]); retinal vasculitis (HR: 9.37 [CI: 1.14-76.75]); unspecified purulent endophthalmitis (HR: 1.50 [CI: 1.07-2.12]); pan-uveitis (HR: 5.87 [CI: 2.24-15.36]); and sympathetic uveitis (HR: 10.26 [CI: 1.28-82.50]) (see **Table 2**).

At the 5-year timepoint, an overall increased risk for uveitis was found (HR: 2.48 [CI: 2.14-2.88]). Furthermore, we also performed a risk-stratification analysis on potential factors associated with uveitis development among users of immune checkpoint inhibitors at the five-year follow-up point. We observed elevated hazard ratio (HR) values for uveitis development among ICI users who were aged less than (HR: 2.91 [CI: 2.37-3.57]) and greater (HR: 2.37 [CI: 1.89-2.96]) than 65 years of age; female (HR: 2.63 [CI: 2.10-3.29]); male (HR: 2.74 [CI: 2.22-3.38]), White ethnicity (HR: 2.99 [CI: 2.51-3.55]), Asian ethnicity (HR: 3.14 [CI: 1.08-9.11]); smokers (HR: 1.79 [CI: 1.03-3.12]) and had comorbidities of hypertension (HR: 1.56 [CI: 1.16-2.10]), dyslipidemia (HR: 1.65 [CI: 1.15-2.36]) and cerebrovascular history (HR: 2.21 [CI: 1.01-4.88]) compared to their respective non-ICI comparators (see **Supplementary Table 6**). The involved uveitis diseases that had an increased risk of association with ICI usage at the 5-year time point were: iridocyclitis (HR: 2.70 [CI: 2.25-3.23]); chorioretinal inflammation (HR: 4.19 [CI: 2.80-6.26]); retinal vasculitis (HR: 13.78 [CI: 1.73-109.80]); unspecified purulent endophthalmitis (HR: 1.62 [CI: 1.20-2.18]); pan-uveitis (HR: 8.44 [CI: 3.49-20.39]); and sympathetic uveitis (HR: 12.35 [CI: 1.55-98.20]) (see **Table 2**).

At the 12-year timepoint, the ICI group was found to have an increased risk for developing uveitis compared to the non-ICI recipient group (HR: 2.39 [CI: 2.07-2.75]) (see **Table 2**). Furthermore, we also performed a risk-stratification analysis on potential factors associated with uveitis development among users of immune checkpoint inhibitors at the twelve-year follow-up time point. We observed elevated hazard ratio (HR) values for developing uveitis among ICI users who were aged less than (HR: 2.80 [CI: 2.31-3.41]) and greater than 65 years of age (HR: 2.24 [CI: 1.80-2.78]), female (HR: 2.54 [CI: 2.05-3.15]) and male genders (HR: 2.51 [CI: 2.05-3.07]), ethnicity of white (HR: 2.70 [CI: 2.29-3.18]) and Asian (HR: 2.78 [CI: 1.02-7.58]), smoker (HR: 1.79 [CI: 1.03-3.12]), as well as the comorbidity of hypertension (HR: 1.56 [CI: 1.17-2.09]) and dyslipidemia (HR: 1.61 [CI: 1.13-2.28]), compared to their respective non-ICI comparators (see **Table 3**). The involved uveitis diseases that had an increased risk of association with ICI usage at the 12-year time point were: iridocyclitis (HR: 2.59 [CI: 2.25-3.23]); chorioretinal inflammation (HR: 3.71 [CI: 2.57-5.37]); retinal vasculitis (HR: 5.64 [CI: 1.54-20.62]); unspecified purulent endophthalmitis (HR: 1.58 [CI: 1.18-2.12]); pan-uveitis (HR: 8.06 [CI: 3.51-18.51]); sympathetic uveitis (HR: 7.57 [CI: 1.55-36.92]) (see **Table 2**).

**Table 3:** Stratification for risk of uveitis exposed to immune checkpoint inhibitors compared to non-immune checkpoint inhibitors in all follow-up duration.

	ICI		Non-ICI		HR (95% C.I.)
	N	No. of uveitis	N	No. of uveitis	
Age					
<65	35018	281	35018	184	2.80 (2.31–3.41)
≥65	36913	206	36913	162	2.24 (1.80–2.78)
Sex					
Female	30879	221	30879	168	2.54 (2.05–3.15)
Male	39326	257	39326	178	2.51 (2.05–3.07)
Race					
White	54992	405	54992	265	2.70 (2.29–3.18)
Black	5932	36	5932	57	1.22 (0.79–1.90)
Asian	2401	10	2401	10	2.78 (1.02–7.58)
Tobacco use	2922	29	2922	24	1.79 (1.03–3.12)
Hypertension	16838	93	16838	109	1.56 (1.17–2.09)
Dyslipidemia	11561	66	11561	76	1.61 (1.13–2.28)
Coronary artery disease	4927	18	4927	19	1.72 (0.88–3.39)
Cerebrovascular disease	2108	16	2108	13	2.10 (0.97–4.53)

If the patient’s count is 1–10, the results indicate a count of 10.

**Figure 1:**
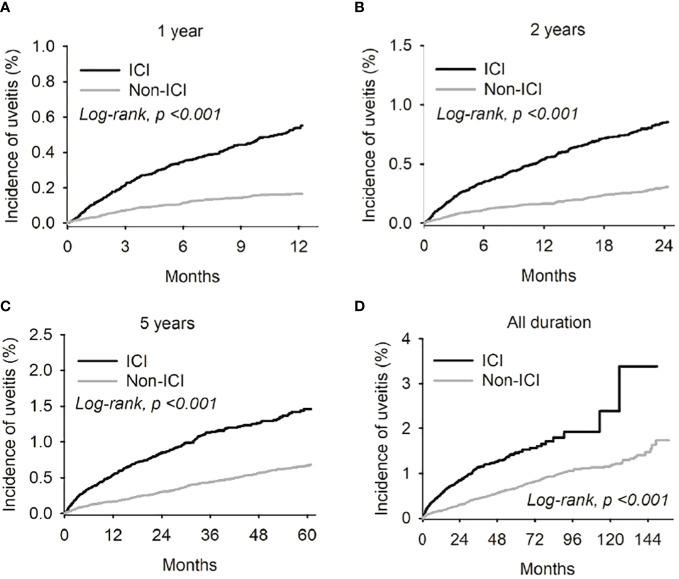
Kaplan-Meier curves were employed to illustrate the risk of uveitis in both the Immune complex inhibitor (ICI) and non-ICI cohorts across different follow-up durations - specifically, at the 1-year **(A)**, 2-year **(B)**, and 3-year **(C)** intervals from the index date. Furthermore, the Kaplan-Meier curve for the cumulative uveitis risk over the entire study period was also presented **(D)**.

The associated Kaplan-Meier curves are shown in **Figure 1**. When stratified by follow-up duration, there was a significant association between new-onset uveitis and receiving ICI therapy after the 1-year follow-up point (p<0.001) (see **Figure 1**).

### 3.3 Risk of uveitis among the different classes of immune check point inhibitors

The risk of uveitis was also assessed across the different classes of ICI. As part of this subgroup analysis, combinatory ICI prescription was compared to monotherapy for uveitis risk (see **Supplementary Table 7**). The various ICI medications and their respective classes analyzed in our study are listed in **Supplementary Table 1** and **Supplementary Figure 2**.

Among recipients of the ICI monotherapy regimens, the class of anti-PD-1 (HR:1.98 [CI: 1.65-2.37]), and anti-CTLA-4 (HR:5.86 [CI:1.99-17.24]) showed elevated hazard ratios associated with uveitis development compared to their non-ICI comparators. However, no increased risk of uveitis was seen among monotherapy recipients of the anti-PD-L1 class (HR: 1.15 [CI:0.72-1.85]) compared to their non-ICI comparators.

Among recipients of the combinatory ICI regimens, cancer patients exposed to a combination of anti-PD-1 and anti-CTLA-4 exhibited elevated hazard ratios associated with uveitis development (HR: 5.04 [CI:3.55-7.16]) compared to their non-ICI comparators. However, those exposed to a combination of anti-PD-1 and anti-PD-L1 did not show an increased risk for uveitis (HR: 2.47 [CI:0.81-7.50]). Unfortunately, insufficient data precluded the analysis of uveitis risk among patients who received a combined regimen of anti-PD-L-1 and anti-CTLA-4.

## 4 Discussion

To the best of our knowledge, our study is one of the first to investigate the association between uveitis and the use of ICI among cancer patients.

### 4.1 Novel findings

Our study revealed an increased risk of uveitis development in cancer patients undergoing treatment with immune checkpoint inhibitors (ICI). This increased uveitis risk persisted throughout the study period following the index date. Notably, elevated hazard ratios were observed for uveitis development among ICI recipients compared to their non-ICI counterparts, encompassing those aged 65 years and older, across all genders, those of white and Asian ethnicities, those with smoking history and among those with comorbid hypertension and dyslipidemia, compared to their non-ICI counterparts. Additionally, there was an increased risk for specific uveitis diseases, such as iridocyclitis, chorioretinal inflammation, retinal vasculitis, unspecified purulent endophthalmitis, pan-uveitis and sympathetic uveitis. As part of a subgroup analysis on the different monotherapy treatment regimes, we also found that the class of anti-PD-1 and anti-CTLA-4 had elevated hazard ratios associated with uveitis development compared to their non-ICI comparators. As part of a subgroup analysis on different combinatory treatment regimes, we also found that the combination of anti-PD-1 and anti-CTLA-4 exhibited elevated hazard ratios associated with uveitis development compared to their non-ICI comparators. However, those exposed to a combination of anti-PD-1 and anti-PD-L1 did not show an increased risk for uveitis compared to their non-ICI comparators.

### 4.2 Clinical implications

Our study represents one of the most extensive investigations into the association between uveitis and ICI usage. This is particularly significant due to the paucity of large-scale studies on this topic. Furthermore, the integration of short-term and long-term timeframes in the study of ICI-associated uveitis risk is scarce in the existing literature. Current guidelines suggest risk-stratifying ICI-related ocular toxicities based on clinical presentations and the number of trace cells in the anterior chamber (19). For example, lower-grade toxicities may warrant observation with a non-urgent referral to an ophthalmologist, while higher-grade toxicities necessitate an urgent ophthalmological evaluation and potential discontinuation of the ICI. Therefore, demonstrating both the short-term (1 year) and long-term (2 years, 5 years, and 12 years) risks of ICI-associated uveitis in a single study not only contributes to the understanding of ICI-associated ophthalmologic manifestations but also emphasize the importance of adopting a cautious, risk-stratifying approach towards ICI usage.

### 4.3 Pathogenesis of ICI with drug-induced uveitis

Autoimmunity is believed to contribute to the development of drug-induced uveitis following ICI (see **Supplementary Figure 2**) treatment. Typically, T-cells play a crucial role in neutralizing foreign invaders. Immune checkpoints regulate these T cells by inducing self-tolerance. Proteins involved in self-tolerance include T-lymphocyte-associated protein 4 (CTLA-4), programmed death-1 (PD-1) and programmed death ligand-1 (PD-L1). Notably, tumor cells bypass these immune defenses by mimicking these proteins. ICI therapy targets cancer cells by reprogramming these immune checkpoints (20). However, the unintended consequence of this immune-related reprogramming can lead to systemic manifestations unrelated to the initially targeted cancer site, termed immune-related adverse events (IRAEs).

### 4.4 Comparison to literature

Our first comparison study of interest was by Anquetil et al. This was a retrospective pharmacovigilance study that reported 211 adverse events related to ICI involving uveitis (21). The median age of those affected was around 45 years, with females being the most commonly affected gender. These findings were partially consistent with our study. However, Chaudot et al. showed equal distribution in gender in terms of ICI-related uveitis (22). The reasons for our study finding an increased risk of uveitis in both male and female ICI users compared to their non-ICI comparators remain unclear and warrant further exploration in future studies.

Additionally, Anquetil et al. suggested that combined prescriptions of different classes of ICIs were associated with uveitis, although Anquetil et al. did not specify the classes involved. In comparison, a study by Chaudot et al. showed half of their ICI-associated uveitis cases occurred with nivolumab (an anti-PD-1 class of ICI) monotherapy (32% of total cases) or ipilimumab/nivolumab (anti-CTLA-4 and anti-PD1 class of ICI) (28% of total cases) combination (22). Notably, our results complemented Chaudot et al., with some key differences. We found that monotherapy with the anti-CTLA-4 class of ICI drugs (see **Supplementary Table 7**) had the highest hazard ratios for uveitis development compared to all other classes. Furthermore, our study also showed that anti-CTLA-4 and anti-PD1 combinatory regimes (see **Supplementary Table 7**) had the highest hazard ratio for uveitis development. We are unsure of the reason behind the differences in data between ours and Chaudot et al. However, our findings were consistent with those of Fang et al. and Sun et al., where they showed the greatest uveitis risk was from monotherapy and combination therapy involving the anti-CTLA-4 class (23, 24). The heterogeneity in data across these studies highlights a need for a more definitive consensus on which ICI is most strongly associated with uveitis. It is important to note that ICI studies frequently rely on adverse event reporting by clinical investigators, rendering them susceptible to potential bias stemming from incomplete reporting or monitoring (25). This susceptibility could have played a role in contributing to the observed heterogeneity in data. Lastly, regarding the mechanisms behind why CTLA-4 class of ICI is most associated with uveitis, it has been hypothesized that this class of drugs’ mechanism of action focused more on the proximal stages of T-cell activation. This selective targeting of the early stages of T-cell activation may theoretically make recipients of anti-CTLA-4 drugs more susceptible to developing autoimmune adverse events like uveitis (23). Further studies are still needed to confirm these hypotheses and findings.

Secondly, Anquetil et al. highlighted that other cancer drugs, such as protein kinase inhibitors and bisphosphonates, were associated with drug-induced uveitis. It is plausible that our participants may have a history of exposure to protein kinase inhibitors as well as bisphosphonate drugs due to their cancer history (26). These potential confounders in our results merit further exploration.

In another study of interest was by Chaudot et al. (22), where 36 patients with ICI-induced adverse drug reactions were recruited. Among these ICI-associated adverse drug reactions, uveitis was found to account for 69% of total cases. Demographically, Chaudot et al. reported an equal distribution between genders, a mean age of 58.5 years old and a predisposition towards Caucasian ethnicity. In our study, we found an increased hazard ratio for uveitis occurrence after ICI usage across both genders, Caucasian and Asian ethnicities and all age groups when compared to their corresponding non-ICI comparators. The predilection of ICI-related uveitis towards white ethnicity was expected in our study, as 76.5% of our study population comprised individuals of white ethnicity. However, we also observed elevated hazard ratios related to uveitis among individuals of Asian ethnicity (see **Table 3**). This finding was intriguing, considering Asians comprised only 3.3% of our study population (See **Table 1**). Certain cancers, such as melanoma and lung cancer, may exhibit varying subtypes in different ethnic groups. For example, the acral lentiginous melanoma subtype is more common in the Asian population than in the white population. These distinct melanoma subtypes prevalent in Asians may harbor unique genetic differences, such as a predilection for BRAF or N-RAS mutations or other chromosomal abnormalities (27). Such genetic distinctions among melanoma subtypes common in the Asian population could lead to differences in antigen presentation and a predisposition toward autoimmune reactions in response to ICI. However, it is important to acknowledge that our study’s small sample size among Asians limits the interpretability of these findings.

Furthermore, it is interesting to note that melanoma diagnosis accounted for 46.4% of all cancer patients from the study population of Chaudot et al. Sun et al. further noted most of the patients who had ICI-associated uveitis from their study had melanoma-associated diagnoses (23). This raises intriguing questions about the impact of different cancer types on uveitis risk. A brief review of the available literature has suggested that melanoma can increase the risk of ocular inflammatory disorders such as uveitis. One hypothesis for such an association between uveitis and melanoma is likely due to the increased immunogenicity among melanoma patients, as hinted in lab studies where increased antiretinal antibodies were found among the serum of melanoma patients (28). The diagnosis of melanoma was, therefore, another potential confounder for our study, as we did not exclude melanoma from our study participants.

Chaudot et al. also reported the average onset of drug-induced uveitis to be 17 weeks. Other studies have also indicated an average onset of uveitis after ICI to be 6.8 ± 5.5 months (29). Our study not only demonstrated a significant association with uveitis among ICI users (p<0.001) after the 1-year period but also stands out as one of the first and largest studies to show such an association over a follow-up period of up to 12 years. We hypothesize that the pro-inflammatory effects induced by ICI usage contribute to the pathophysiology underlying the extended duration of uveitis risk among ICI users.

Regarding uveitis subtypes, Chaudot et al. showed that anterior uveitis was the most common subtype affected (44%), followed by pan-uveitis (28%) and posterior uveitis (19%). These findings were consistent with other studies (20, 29) and ours. In our study, we observed an overall increased uveitis risk, specifically for the ICD-10 categories of iridocyclitis (HR: 2.70 [CI: 2.25-3.23]); chorioretinal inflammation (HR: 4.19 [CI: 2.80-6.26]); retinal vasculitis (HR: 13.78 [CI: 1.73-109.80]); unspecified purulent endophthalmitis (HR: 1.62 [CI: 1.20-2.18]); pan-uveitis (HR: 8.44 [CI: 3.49-20.39]); and sympathetic uveitis (HR: 12.35 [CI: 1.55-98.20]).

On a related note, Vogt-Koyanagi-Harada (VKH) syndrome is a rare inflammatory condition characterized by bilateral, granulomatous panuveitis formation. Sensitization to melanin-related antigens has been proposed as a mechanism behind such a rare syndrome, where autoimmune cross-reaction between melanoma-related cancer cells and normal choroidal melanocytes is thought to occur (23). Previous studies have linked VKH with ICI agents such as nivolumab, ipilimumab and pembrolizumab (30–32). However, studies on VKH have mostly been limited to case reports. It would have been interesting to assess the risk of such an association with ICI from a population-based study like ours. Unfortunately, due to insufficient case numbers, such statistical analysis was not possible from within our study. The rarity of VKH may have contributed to the lack of data from our study. However, it is impossible to rule out other factors that may have influenced our results, including HLA types. In one study, 100% of patients with VKH-associated uveitis who underwent HLA typing were found to have HLA-DRB1*04:05. DRB1*04:05 is one of the most common alleles that can lead to increased risk for VKH (33). As our study was retrospective and based on diagnostic codes, we could not investigate the presence or absence of such risk alleles in our study population.

With regards to the analysis of baseline factors and comorbidities, we identified an association between smoking status and the comorbidities of hypertension, dyslipidemia, and cerebrovascular comorbidities with the risk of uveitis. These associations may be attributed to specific inflammatory markers upregulated in patients with such baseline factors. For instance, interleukin (IL) 23 has been found to be elevated in smokers and patients with dyslipidemia (34, 35). This is interesting as IL-23 is a pro-inflammatory cytokine that plays an active role in activating T-helper 17 (Th17) cells. This activation of Th17 cells can trigger the secretion of additional pro-inflammatory cytokines, such as IL-17, as part of the broader IL-17/IL-23 signaling pathway (36, 37). The Interleukin-23/interleukin-17 (IL-23/Il-17) axis has been implicated in various immune-mediated inflammatory diseases, as demonstrated in animal model studies. This pathway is central to uveitis formation and is theorized to result from damage to retinal pigment epithelium (RPE) cells.

Under normal circumstances, the RPE plays a crucial role in maintaining the immune-privileged status of the eye. This is achieved through its contribution to the structure of the blood-retinal barrier and its regulatory effect on homeostatic inflammatory processes, such as keeping the T cells in an anergic state. Disruption of the RPE caused by elevated inflammatory cytokines (like IL-17 or IL-23), as observed in patients with dyslipidemia, can activate other downstream inflammatory pathways (e.g. NF-kb signaling pathway). This cascade may lead to the recruitment of additional inflammatory mediators, ultimately resulting in uveitis formation. If the proposed pathophysiology involving the IL-17/IL-23 signaling pathway holds true for uveitis and certain cardiovascular comorbidities, it raises intriguing questions about the potential therapeutic implications of targeting the IL-17/IL-23 axis. Notably, therapies directed at these pathways have demonstrated success in treating diseases such as psoriasis, psoriatic arthritis and Crohn’s disease (38, 39), owing to the similarly shared IL-17/IL-23 signaling pathways from among these conditions. Whether these therapies could prevent uveitis requires further exploration.

## 5 Strength and limitations

The strength of our study lies in the utilization of a large population-based electronic health records database. Furthermore, we employed ICD-10, HCPCS and RXNORM diagnostic codes in order to recruit cancer patients who are also recipients of ICI therapy. Our study further benefited from propensity score matching, longitudinal design, and extended follow-up duration. Additionally, we enhanced the validity of our study by excluding other confounding conditions related to uveitis (6, 8, 14, 40–42)

Our study has several limitations that should be considered. Firstly, there is the possibility of residual confounding, including factors such as the use of bisphosphonates and protein kinase inhibitors, as well as the implications of the different cancer subtypes, such as melanoma, on our results. Additionally, a history of intraocular surgery could be a potential factor (43). Moreover, the generalizability of our findings may be limited as a substantial proportion (76.5%) of our ICI-receiving study participants are of white ethnicity, and ethnicity-related differences have been shown to influence uveitis presentations and outcomes (44, 45).

Secondly, our data analysis lacks information on dosage, which may have influenced the observed uveitis risk in ICI patients. Some studies have suggested that autoimmune adverse events from certain ICI medications are dose-dependent (46). This is a topic of which future studies can further explore.

Another limitation is related to our restricted outcomes of interest. Our electronic health registry codes only allowed us to capture the first new onset of uveitis after ICI usage, and we cannot comment on uveitis reoccurrence. Studies, such as the one by Qian et al., have shown that ocular IRAE can reoccur even after discontinuation of ICI use (29). The theory is that the history of ICI use might upregulate the immune response in such patients, creating an extended pro-inflammatory state and increasing the risk of uveitis recurrence. These factors warrant future exploration in future studies.

## 6 Conclusions

In summary, our study revealed an increased risk for uveitis among cancer patients receiving ICI after a one-year follow-up. Our findings further demonstrated an increased hazard ratio for uveitis development among ICI recipients aged below 65 years and those aged 65 years and older, across all genders, those of white and Asian ethnicities, smokers, and those with comorbid hypertension and dyslipidemia, compared to their non-ICI counterparts. The specific uveitis-related diseases at risk were iridocyclitis, chorioretinal inflammation, retinal vasculitis, unspecified purulent endophthalmitis, pan-uveitis and sympathetic uveitis. While ocular complications such as drug-induced uveitis from ICI are infrequent, increased awareness remains imperative for guiding the use of these novel agents among cancer patients at risk.

## Data availability statement

The raw data supporting the conclusions of this article will be made available by the authors, without undue reservation.

## Ethics statement

The study adhered to the tenets of the Declaration of Helsinki. Institutional Review Board approval was not required because deidentified data were used for this retrospective analysis. As the TriNetX database has been accredited by an expert to be HIPPA-compliant as well as comprised of only de identified data, ethics approval was not required.

## Author contributions

HK: Conceptualization, Data curation, Formal Analysis, Funding acquisition, Investigation, Methodology, Project administration, Resources, Software, Supervision, Validation, Visualization, Writing – original draft, Writing – review & editing. CC: Conceptualization, Data curation, Formal Analysis, Methodology, Project administration, Supervision, Validation, Writing – original draft, Writing – review & editing. AH: Conceptualization, Data curation, Formal Analysis, Investigation, Methodology, Project administration, Software, Supervision, Validation, Writing – original draft, Writing – review & editing. YW: Conceptualization, Data curation, Investigation, Methodology, Software, Supervision, Writing – original draft, Writing – review & editing. CL: Conceptualization, Data curation, Formal Analysis, Investigation, Methodology, Project administration, Software, Supervision, Validation, Writing – original draft, Writing – review & editing. NH: Conceptualization, Data curation, Investigation, Methodology, Software, Supervision, Writing – original draft, Writing – review & editing. Y-YT: Conceptualization, Data curation, Formal Analysis, Investigation, Methodology, Project administration, Software, Supervision, Validation, Writing – original draft, Writing – review & editing. JW: Funding acquisition, Investigation, Methodology, Project administration, Resources, Software, Validation, Visualization, Writing – original draft, Writing – review & editing, Conceptualization, Data curation, Formal Analysis.
