# Supplementary material for: Association between immune checkpoint inhibitor medication and uveitis: a population-based cohort study utilizing TriNetX database
Source: Front Immunol. 2024 Jan 9;14:1302293. doi: 10.3389/fimmu.2023.1302293 (PMC10803449; doi:10.3389/fimmu.2023.1302293)
Supplement: Supplementary file 1 [file DataSheet_1.pdf]

SUPPLEMENTARY MATERIAL:

Category	Code	Content
**Immune checkpoint inhibitors**		
Programmed death-1 (PD-1) inhibitors		
Medication	RXNORM:1597876	Nivolumab
Procedure	HCPCS:J9299	Injection, nivolumab, 1 mg
Medication	RXNORM:2058826	Cemiplimab
Procedure	HCPCS:J9119	Injection, cemiplimab-rwlc, 1 mg
Medication	RXNORM:2539967	Dostarlimab
Procedure	HCPCS:J9272	Injection, dostarlimab-gxly, 10 mg
Medication	RXNORM:1547545	Pembrolizumab
Procedure	HCPCS:J9271	Injection, pembrolizumab, 1 mg
Programmed death ligand-1 inhibitors (PD-L1)		
Medication	RXNORM:1792776	Atezolizumab
Procedure	HCPCS:J9022	Injection, atezolizumab, 10 mg
Medication	RXNORM:1875534	Avelumab
Procedure	HCPCS:J9023	Injection, avelumab, 10 mg
Medication	RXNORM:1919503	Durvalumab
Procedure	HCPCS:J9173	Injection, durvalumab, 10 mg
Cytotoxic T-lymphocyte antigen 4 (CTLA-4)		
Medication	RXNORM:1094833	Ipilimumab
Procedure	HCPCS:J9228	Injection, ipilimumab, 1 mg
**Cancer**		
Diagnosis	ICD-10-CM: C00–C14	Malignant neoplasms of lip, oral cavity and pharynx
Diagnosis	ICD-10-CM: C15–C26	Malignant neoplasms of digestive organs
Diagnosis	ICD-10-CM: C30–C39	Malignant neoplasms of respiratory and intrathoracic organs
Diagnosis	ICD-10-CM: C40–C41	Malignant neoplasms of bone and articular cartilage
Diagnosis	ICD-10-CM: C43–C44	Melanoma and other malignant neoplasms of skin
Diagnosis	ICD-10-CM: C45–C49	Malignant neoplasms of mesothelial and soft tissue
Diagnosis	ICD-10-CM: C50–C50	Malignant neoplasms of breast
Diagnosis	ICD-10-CM: C51–C58	Malignant neoplasms of female genital organs
Diagnosis	ICD-10-CM: C60–C63	Malignant neoplasms of male genital organs
Diagnosis	ICD-10-CM: C64–C68	Malignant neoplasms of urinary tract
Diagnosis	ICD-10-CM: C69–C72	Malignant neoplasms of eye, brain and other parts of central nervous system
Diagnosis	ICD-10-CM: C73–C75	Malignant neoplasms of thyroid and other endocrine glands
Diagnosis	ICD-10-CM: C76–C80	Malignant neoplasms of ill-defined, other secondary and unspecified sites
Diagnosis	ICD-10-CM: C7A	Malignant neuroendocrine tumors
Diagnosis	ICD-10-CM: C7B	Secondary neuroendocrine tumors
Diagnosis	ICD-10-CM: C81–C96	Malignant neoplasms of lymphoid, hematopoietic and related tissue

Supplementary Table 1: ICD definitions for immune checkpoint inhibitors and cancer recruited for this study

Exclusion:

Category	Code	Content
Diagnosis	ICD-10-CM: B20	Human immunodeficiency virus [HIV] disease
Diagnosis	ICD-10-CM: R75	Inconclusive laboratory evidence of human immunodeficiency virus [HIV]
Diagnosis	ICD-10-CM: Z21	Asymptomatic human immunodeficiency virus [HIV] infection status
Diagnosis	ICD-10-CM: B97.35	Human immunodeficiency virus, type 2 [HIV 2] as the cause of diseases classified elsewhere
Diagnosis	ICD-10-CM: A51	Early syphilis
Diagnosis	ICD-10-CM: A52.7	Other symptomatic late syphilis
Diagnosis	ICD-10-CM: A52.75	Syphilis of kidney and ureter
Diagnosis	ICD-10-CM: D86	Sarcoidosis
Diagnosis	ICD-10-CM: D89.89	Other specified disorders involving the immune mechanism, not elsewhere classified
Diagnosis	ICD-10-CM: M35.2	Behçet's disease
Diagnosis	ICD-10-CM: K75.4	Autoimmune hepatitis
Diagnosis	ICD-10-CM: L40	Psoriasis
Diagnosis	ICD-10-CM: L41	Parapsoriasis
Diagnosis	ICD-10-CM: M32	Systemic lupus erythematosus
Diagnosis	ICD-10-CM: M05	Rheumatoid arthritis with rheumatoid factor
Diagnosis	ICD-10-CM: M06	Other rheumatoid arthritis
Diagnosis	ICD-10-CM: M08	Juvenile arthritis
Diagnosis	ICD-10-CM: N18	Chronic kidney disease

Supplementary Table 2: Exclusion diagnoses

**Outcome:**
ICD-10-CM: H20 Iridocyclitis
ICD-10-CM: H30 Chorioretinal inflammation
ICD-10-CM: H35.06 Retinal vasculitis
ICD-10-CM: H44.00 Unspecified purulent endophthalmitis
ICD-10-CM: H44.11 Panuveitis
ICD-10-CM: H44.13 Sympathetic uveitis
ICD-10-CM: H20.823 Vogt-Koyanagi syndrome, bilateral
ICD-10-CM: H30.819 Harada's disease, unspecified eye

Supplementary Table 3: Outcome defintions.

	ICI		Non-ICI		
	N	No. of uveitis	N	No. of uveitis	HR (95% C.I.)
Age					
<65	35018	174	35018	51	3.73 (2.73–5.10)
≥65	36913	138	36913	47	3.24 (2.33–4.52)
Sex					
Female	30879	145	30879	56	2.84 (2.08–3.86)
Male	39326	161	39326	53	3.34 (2.45–4.56)
Race					
White	54992	257	54992	75	3.79 (2.93–4.90)
Black	5932	21	5932	17	1.37 (0.72–2.60)
Asian	2401	10	2401	10	3.82 (0.79–18.43)
Tobacco use	2922	23	2922	13	2.01 (1.02–3.98)
Hypertension	16838	65	16838	38	1.94 (1.30–2.89)
Dyslipidemia	11561	49	11561	24	2.26 (1.39–3.68)
Coronary artery disease	4927	13	4927	10	2.27 (0.90–5.69)
Cerebrovascular disease	2108	14	2108	10	3.94 (1.30–11.99)

If the patient's count is 1–10, the results indicate a count of 10.

Supplementary Table 4. Stratification for risk of uveitis exposed to immune checkpoint inhibitors compared to non-immune checkpoint inhibitors in one year follow-up duration

	ICI		Non-ICI		
	N	No. of uveitis	N	No. of uveitis	HR (95% C.I.)
Age					
<65	35018	223	35018	78	3.50 (2.70–4.53)
≥65	36913	180	36913	82	2.77 (2.13–3.60)
Sex					
Female	30879	184	30879	78	2.89 (2.22–3.78)
Male	39326	212	39326	81	3.22 (2.49–4.17)
Race					
White	54992	333	54992	121	3.41 (2.76–4.20)
Black	5932	30	5932	30	1.32 (0.79–2.20)
Asian	2401	10	2401	10	4.70 (0.99–22.31)
Tobacco use	2922	26	2922	17	1.87 (1.01–3.46)
Hypertension	16838	78	16838	63	1.60 (1.15–2.24)
Dyslipidemia	11561	55	11561	41	1.67 (1.11–2.50)
Coronary artery disease	4927	17	4927	11	2.14 (1.00–4.60)
Cerebrovascular disease	2108	15	2108	10	2.66 (1.08–6.57)

If the patient's count is 1–10, the results indicate a count of 10.

Supplementary Table 5. Stratification for risk of uveitis exposed to immune checkpoint inhibitors compared to non-immune checkpoint inhibitors in 2 years follow-up duration

	ICI		Non-ICI		
	N	No. of uveitis	N	No. of uveitis	HR (95% C.I.)
Age					
<65	35018	272	35018	146	2.91 (2.37–3.57)
≥65	36913	204	36913	130	2.37 (1.89–2.96)
Sex					
Female	30879	215	30879	127	2.63 (2.10–3.29)
Male	39326	252	39326	138	2.74 (2.22–3.38)
Race					
White	54992	394	54992	199	2.99 (2.51–3.55)
Black	5932	36	5932	46	1.30 (0.83–2.04)
Asian	2401	10	2401	10	3.14 (1.08–9.11)
Tobacco use	2922	29	2922	23	1.79 (1.03–3.12)
Hypertension	16838	91	16838	93	1.56 (1.16–2.10)
Dyslipidemia	11561	65	11561	61	1.65 (1.15–2.36)
Coronary artery disease	4927	18	4927	17	1.73 (0.88–3.41)
Cerebrovascular disease	2108	16	2108	11	2.21 (1.01–4.88)

If the patient's count is 1–10, the results indicate a count of 10.

Supplementary Table 6 Stratification for risk of uveitis exposed to immune checkpoint inhibitors compared to non-immune checkpoint inhibitors in 5 years follow-up duration

	ICI		Non-ICI		
	N	No. of uveitis	N	No. of uveitis	HR (95% C.I.)
PD-1 inhibitors alone	50239	270	50239	259	1.98 (1.65–2.37)
PD-L1 inhibitors alone	9814	30	9814	54	1.15 (0.72–1.85)
CTLA-4 alone	1445	20	1445	10	5.86 (1.99–17.24)
PD-1 combined with PD-L1 alone	1315	10	1315	10	2.47 (0.81–7.50)
PD-1 combined with CTLA-4 alone	7633	144	7633	44	5.04 (3.55–7.16)

If the patient's count is 1–10, the results indicate a count of 10.

PD-1: Programmed death-1.

PD-L1: Programmed death ligand-1 inhibitors.

CTLA-4: Cytotoxic T-lymphocyte antigen 4.

Supplementary Table 7 Risk of uveitis and classes of immune checkpoint inhibitors

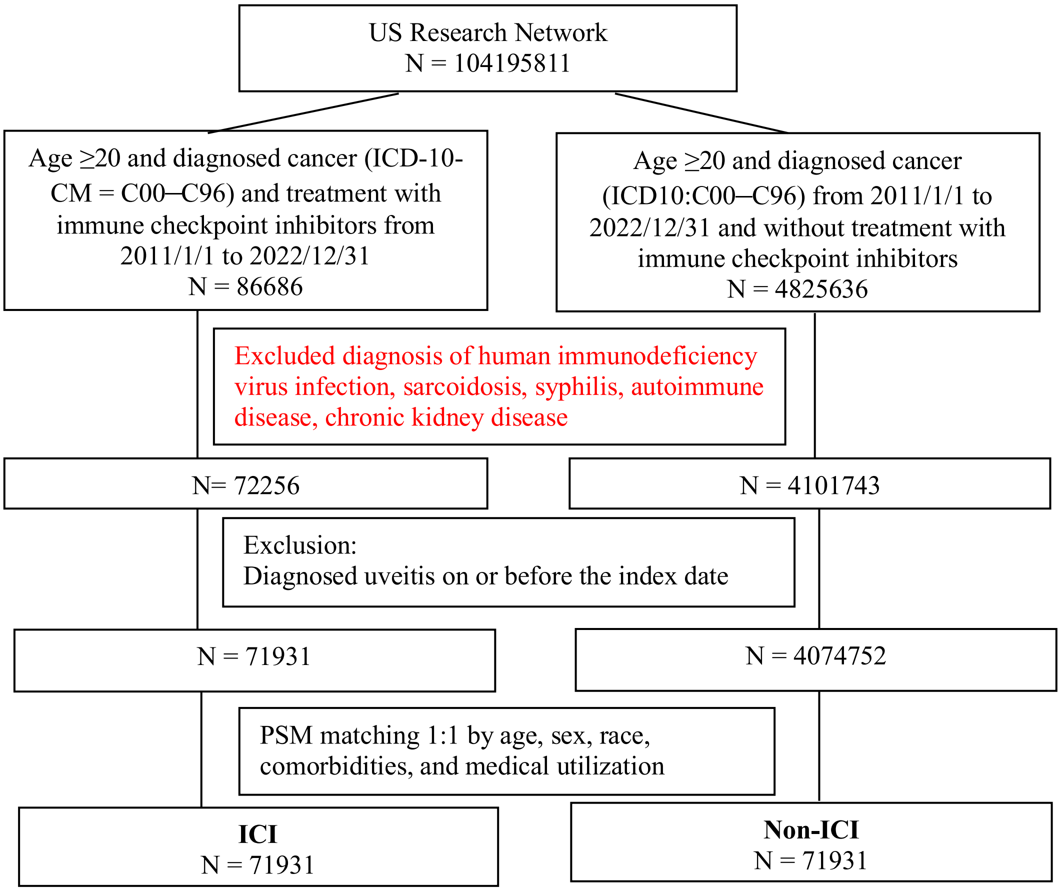

Supplementary Figure 1 Flow-chart of patient selection.

ICI, Immune Complex Inhibitors; PSM, Propensity-score matching.

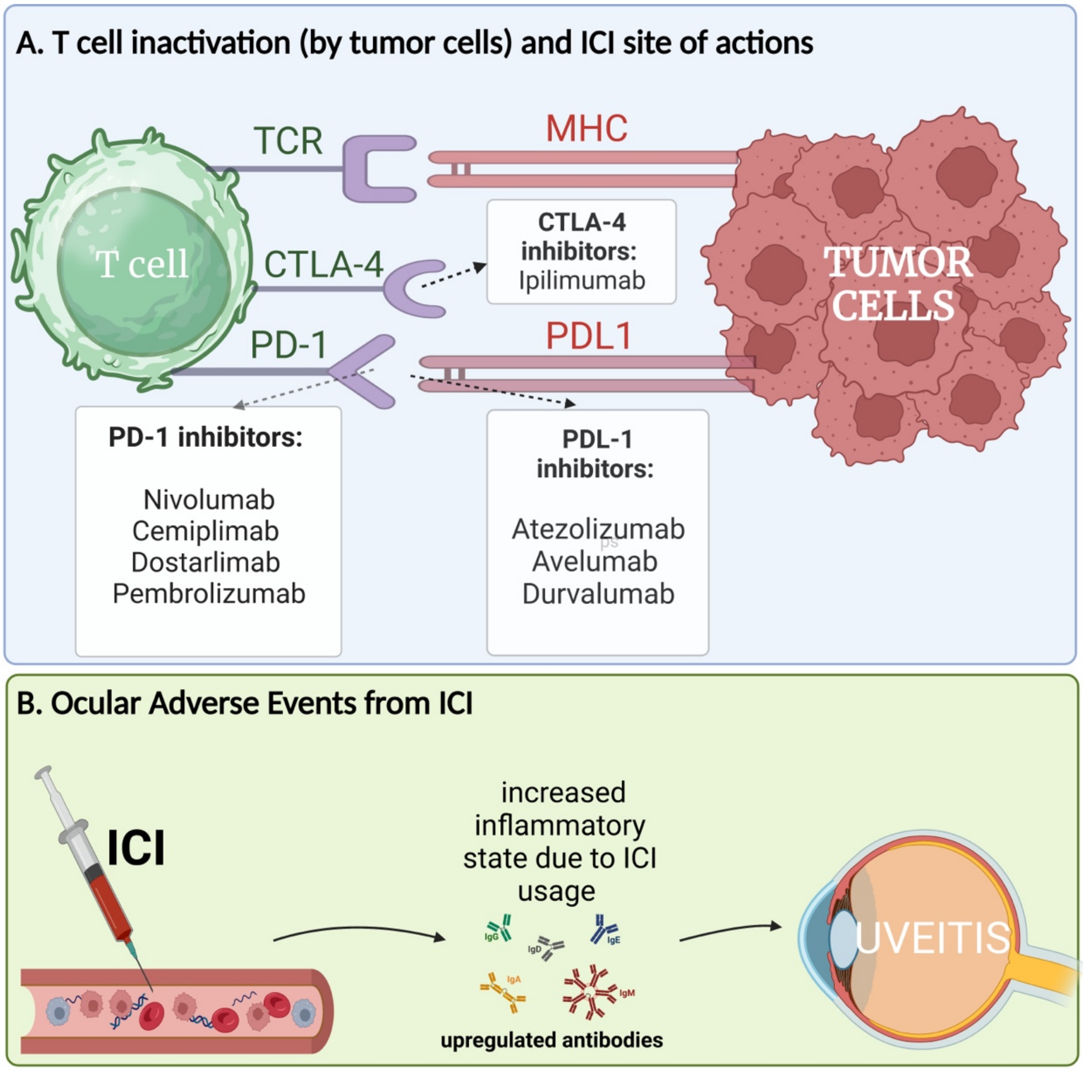

Supplemental Figure 2. Overview of the tumor cell interaction with T cells and the sites of actions of ICI. (A) Tumor cells can inactivate T cells through interactions with certain protein receptors on the T cell surface. Listed are also the site of actions of various immune complex inhibitors (ICI) investigated in this study. (B) ICI can also initiate immune response that affects unintended sites like the eye. This figure was created by an author (A.Y.H) using BioRender (https://app.biorender.com).

Abbreviation: CTLA-4, cytotoxic t-lymphocyte associated antigen; ICI, immune complex inhibitors; MHC, major histocompatibility complex; PD-1, programmed death 1; PDL-1, programmed death ligand 1
